# New Computational Approaches to the Analysis of Single Crystal Diffuse Scattering

**DOI:** 10.1063/4.0001101

**Published:** 2025-10-27

**Authors:** Raymond Osborn, Stephan Rosenkranz, Matthew Krogstad

**Affiliations:** Argonne National Laboratory, Lemont, IL, USA

## Abstract

The power of single crystal diffuse scattering in probing the role of inhomogeneity in material properties has long been recognized by the crystallography community [1]. The method is sensitive to three-dimensional structural correlations over length scales of 5 to 200Å or more, from local relaxations around point defects to nanoscale short-range order. However, in the past, experimental and computational challenges have hindered its widespread adoption as a tool for characterizing disordered materials. With recent advances in both neutron and x-ray instrumentation, it is now routinely possible to measure large volumes of reciprocal space, containing hundreds and often thousands of Brillouin zones, on time scales ranging from a few minutes with synchrotron x-rays to a few hours with neutrons. Such speeds enable diffuse scattering data to be collected as a function of temperature and composition, allowing the evolution of structural fluctuations to be tracked across entire phase diagrams in a matter of days. I will highlight a number of studies where new computational approaches enabled by recent experimental progress have been developed to reveal new insights into the role of structural inhomogeneity, particularly in correlated electron materials [2]. For example, unsupervised machine learning has been used to cluster data voxels according to their common temperature dependences, characterizing both order parameters and fluctuations such as Goldstone Modes and Bragg Glass correlations [3, 4], while 3D-ΔPDF analysis has provided new ways of determining the character and length scale of the structural response to electronic phase transitions, such as charge- density-waves and metal-insulator transitions [5, 6]. Finally, I will also discuss the computational framework that we have developed in order to facilitate data analysis in real-time during measurements [7].
